# What is the appropriate genetic testing criteria for breast cancer in the Chinese population?—Analysis of genetic and clinical features from a single cancer center database

**DOI:** 10.1002/cam4.5976

**Published:** 2023-04-25

**Authors:** Mengqian Ni, Fang Wang, Anli Yang, Qiong Shao, Cong Xue, Wen Xia, Fei Xu, Xi Lin, Jiajia Huang, Xiwen Bi, Ruoxi Hong, Meiting Chen, Qiufan Zheng, Kuikui Jiang, Xinhua Xie, Jun Tang, Xi Wang, Zhongyu Yuan, Shusen Wang, Yanxia Shi, Xin An

**Affiliations:** ^1^ State Key Laboratory of Oncology in South China, Collaborative Innovation Center for Cancer Medicine Sun Yat‐sen University Cancer Center Guangzhou China; ^2^ Department of Medical Oncology Sun Yat‐sen University Cancer Center Guangzhou China; ^3^ Department of Molecular Diagnostics Sun Yat‐sen University Cancer Center Guangzhou China; ^4^ Department of Breast Oncology Sun Yat‐sen University Cancer Center Guangzhou China; ^5^ Department of Ultrasound Sun Yat‐sen University Cancer Center Guangzhou China

**Keywords:** *BRCA1/2* genes, genetic testing criteria, hereditary breast cancer, multigene panel testing, non‐*BRCA* genes, pathogenic or likely pathogenic variants

## Abstract

**Background:**

Genetic testing plays an important role in guiding screening, diagnosis, and precision treatment of breast cancer (BC). However, the appropriate genetic testing criteria remain controversial. The current study aims to facilitate the development of suitable strategies by analyzing the germline mutational profiles and clinicopathological features of large‐scale Chinese BC patients.

**Methods:**

BC patients who had undergone genetic testing at the Sun Yat‐sen University Cancer Center (SYSUCC) from September 2014 to March 2022 were retrospectively reviewed. Different screening criteria were applied and compared in the population cohort.

**Results:**

A total of 1035 BC patients were enrolled, 237 pathogenic or likely pathogenic variants (P/LPV) were identified in 235 patients, including 41 out of 203 (19.6%) patients tested only for *BRCA1/2* genes, and 194 out of 832 (23.3%) received 21 genes panel testing. Among the 235 P/LPV carriers, 222 (94.5%) met the NCCN high‐risk criteria, and 13 (5.5%) did not. While using Desai's criteria of testing, all females diagnosed with BC by 60 years and NCCN criteria for older patients, 234 (99.6%) met the high‐risk standard, and only one did not. The 21 genes panel testing identified 4.9% of non‐*BRCA* P/LPVs and a significantly high rate of variants of uncertain significance (VUSs) (33.9%). The most common non‐*BRCA* P/LPVs were *PALB2* (11, 1.3%), *TP53* (10, 1.2%), *PTEN* (3, 0.4%), *CHEK2* (3, 0.4%), *ATM* (3, 0.4%), *BARD1* (3, 0.4%), and *RAD51C* (2, 0.2%). Compared with *BRCA1/2* P/LPVs, non‐*BRCA* P/LPVs showed a significantly low incidence of NCCN criteria listed family history, second primary cancer, and different molecular subtypes.

**Conclusions:**

Desai's criteria might be a more appropriate genetic testing strategy for Chinese BC patients. Panel testing could identify more non‐*BRCA* P/LPVs than *BRCA1/2* testing alone. Compared with *BRCA1/2* P/LPVs, non‐*BRCA* P/LPVs exhibited different personal and family histories of cancer and molecular subtype distributions. The optimal genetic testing strategy for BC still needs to be investigated with larger continuous population studies.

## 1 INTRODUCTION

Breast cancer (BC) has become the most common cancer globally.^1^ China has the largest number of BC cases, which accounts for 18.4% (416,000) of the global ones.^2^ Approximately 15%–20% of BC have a family history, and 5%–10% are considered to have hereditary breast cancer (HBC).^3^ Due to the increasing influence of genetic factors on BC surveillance, prevention, and treatment decision, genetic testing is rapidly expanding in clinical practice.^4^ Besides the most common *BRCA1/2* genes, HBC‐associated genes are emerging, including other high to moderate penetrance genes such as *PALB2*, *TP53*, *PTEN*, *STK11*, *CDH1*, *CHEK2*, *ATM*, and a variety of low penetrance genes.^5^, ^6^ However, the criteria of germline testing for BC remain controversial. The National Comprehensive Cancer Network (NCCN) criteria recommend genetic testing only for high‐risk patients, which may miss half of the cases who do not meet this standard.^7^, ^8^ Other guidelines such as the American Society of Breast Surgeons (ASBrS) propose genetic testing for all BC patients, which would detect more variants at the cost of testing a large number of patients.^9^, ^10^, ^11^ Especially in the population unfit for the NCCN criteria, the frequency of variants in high‐risk BC genes is only 0.8%.^8^ Therefore, this universal testing strategy will undoubtedly increase the cost and burden of genetic testing. Another important issue is which genes should be detected. Compared to testing for *BRCA1/2* alone, multigene panel testing can improve the detection rate of HBC.^12^ However, a diversity of gene panels were applied in different studies, varying from 6 to more than 100 genes.^13^, ^14^ The expanded panels contain many genes with low penetrance or even unrelated to BC, which will lead to a series of problems, including excessive patient anxiety, difficulty in variants interpretation, and unnecessary screening and prevention strategies.^8^, ^15^

As a heterogeneous group of diseases, different subtypes of BC might have different genetic backgrounds and characteristics.^16^ Current studies mainly focus on HER2‐negative patients, especially triple‐negative breast cancer (TNBC), while the genetic profile of HER2‐positive BC is unclear.^17^ Moreover, there are significant racial differences in HBC.^18^ As the largest BC country, little genetic data concerning BC in the Chinese population is disclosed. Our current study aims to analyze the clinicopathological features and genetic data in a large cohort of Chinese patients diagnosed with HBC, which will facilitate making suitable criteria for germline testing of HBC.

## 2 MATERIALS AND METHODS

### 2.1 Study population

BC patients received germline counseling and testing at the Sun Yat‐sen University Cancer Center (SYSUCC) from September 2014 to March 2022 were retrospectively analyzed via electronic medical record review. Variables obtained included age of cancer diagnosis, personal and family history of cancer, histopathological characteristics, and genetic testing profiles. The criteria for high‐risk BC are as follows: (1) diagnosed with BC at the age ≤45 years; (2) TNBC diagnosed at the age ≤60 years; (3) multiple primary BCs; (4) male BC; (5) ≥1 close blood relative with *BRCA*‐related cancer, including BC, ovarian cancer (OC), pancreatic cancer (PaC), and prostate cancer (PrC).^19^ All patients signed informed consent for genetic testing. The study was approved by the Ethical Committee of SYSUCC and the Ministry of Science and Technology for human genetic resource collection.

### 2.2 Genetic testing

Ethylenediaminetetraacetic acid (EDTA)‐anticoagulated peripheral blood samples from all patients were collected. Germline genomic DNA (gDNA) fragments were isolated from peripheral mononuclear blood cells for genetic testing. Before 2017, testing was limited to *BRCA1/2* variants only. After that, a panel containing 21 genes was extensively applied, including: *BRCA1*, *BRCA2*, *TP53*, *PALB2*, *STK11*, *CDH1*, *PTEN*, *RAD51C*, *CHEK2*, *ATM*, *BRIP1*, *RAD50, BARD1*, *MUTYH*, *MRE11A*, *NBN*, *MLH1*, *MSH2*, *MSH6*, *PMS1*, and *PMS2*. The stratification of 21 genes panel testing was summarized in Table S1. Genetic testing was carried out at the Molecular Diagnostics Department of SYSUCC using BGISEQ‐2000 Sequencing System (BGI).

### 2.3 Genetic variant classification and analysis

Variants were named based on the rules suggested by the Human Genome Variation Society (HGVS) (http://varnomen.hgvs.org/) according to the criteria developed by the International Agency for Research on Cancer (IARC) and the American College of Medical Genetics and Genomics (ACMG).^20^, ^21^ The detected genetic variants were classified into five categories: benign variant (class I), likely benign variant (class II), variant of uncertain significance (VUS, class III), likely pathogenic variant (LPV, class IV), and pathogenic variant (PV, class V). Several databases were used to identify and classify the pathogenicity of genetic variants, such as Clin Var (https://www.clinicalgenome.org/data‐sharing/clinvar/), Leiden Open Variation Database (LOVD) (https://github.com/LOVDnl), BRCA Exchange (https://brcaexchange.org/), and Human Gene Mutation Database (HGMD) (http://www.hgmd.cf.ac.uk/ac/index.php). Pathogenic and likely pathogenic variants (P/LPVs) were defined together as deleterious variants for analysis.

### 2.4 Statistical analysis

The genetic and clinicopathologic characteristics of the enrolled patients were summarized by descriptive statistics. T‐tests were used for continuous variables. Chi‐square tests or Fisher's exact tests were used to compare the categorical variables between P/LPV carriers and non‐P/LPV carriers, and between subgroups of different P/LPV carriers. All *p* values were two‐sided, and *p* values <0.05 was considered statistically significant. Statistical analysis was performed using IBM SPSS Statistics (V25; SPSS).

## 3 RESULTS

### 3.1 Clinicopathological characteristics of the study population

**FIGURE 1:**
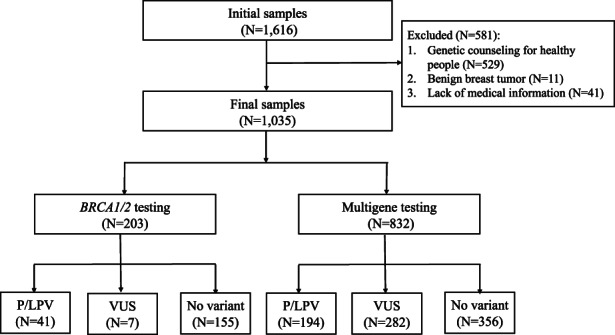
The flowchart of screening and genetic testing for patients.

The flowchart of screening and genetic testing for patients was shown in Figure 1. After reviewing 1616 participants, 1035 pathologically confirmed BC patients were finally enrolled in the analysis. The clinicopathologic characteristics of enrolled patients were summarized in Table 1. All the patients were Han Chinese, and 13 (1.3%) were male gender. The median age at diagnosis of BC was 41 years (range, 21–80 years), with 447 (43.2%) diagnosed before 40 years old. The most common pathological subtype was invasive ductal carcinoma (IDC) (969, 93.6%), followed by ductal carcinoma in situ (DCIS) (35, 3.4%) and invasive lobular carcinoma (ILC) (16, 1.6%). TNBC accounted for 31.1% (322) of patients and HER2‐positive diseases for another 17.8% (184). 238 (23.0%) patients reported having a family history of *BRCA*‐related cancer, including 201 (19.4%) with BC and 37 (3.6%) with OC, PaC, and PrC. 146 (14.1%) patients had other cancer family histories. 85 (8.2%) patients had synchronous or metachronous bilateral BC. 74 (7.2%) patients had other primary cancers, including 70 with secondary primary and 4 with third primary cancers.

**TABLE 1:** Clinicopathological characteristics of the study population.

Variables	Total cohort (*N* = 1035)	P/LPV carriers (*N* = 235)	VUS carriers (*N* = 289)	Non‐variant carriers (*N* = 511)	*P*_*1*_	*P*_*2*_
Gender, *n* (%)					0.269	0.296
Male	13 (1.3)	4 (1.7)	5 (1.7)	4 (0.8)		
Female	1022 (98.7)	231 (98.3)	284 (98.3)	507 (99.2)		
Age at diagnosis, Median (Range)	41 (21, 80)	40 (24, 78)	41 (22, 71)	42 (21, 80)	**0.004**	0.353
Age group, *n* (%)					0.182	0.710
≤30 years	90 (8.7)	26 (11.0)	22 (7.6)	42 (8.2)		
31–40 years	357 (34.5)	90 (38.3)	105 (36.3)	162 (31.7)		
41–50 years	348 (33.6)	70 (29.8)	93 (32.2)	185 36.2)		
51–60 years	180 (17.4)	38 (16.2)	51 (17.7)	91 (17.8)		
≥60 years	60 (5.8)	11 (4.7)	18 (6.2)	31 (6.1)		
Histological subtype, *n* (%)					**0.029**	>0.999
IDC	969 (93.6)	227 (96.6)	268 (92.7)	474 (92.7)		
DCIS	35 (3.4)	2 (0.9)	12 (4.2)	21 (4.1)		
ILC	16 (1.6)	5 (2.1)	4 (1.4)	7 (1.4)		
Special type^b^	15 (1.4)	1 (0.4)	5 (1.7)	9 (1.8)		
Histological grade, *n* (%)					**0.014**	0.972
G1 + G2	459 (44.3)	94 (40.0)	133 (46.0)	232 (45.4)		
G3	512 (49.5)	134 (57.0)	135 (46.7)	243 (47.5)		
Unknown	64 (6.2)	7 (3.0)	21 (7.3)	36 (7.1)		
HR status, *n* (%)					0.697	0.332
Negative	378 (36.5)	85 (36.2)	99 (34.3)	194 (38.0)		
Positive	657 (63.5)	150 (63.8)	190 (65.7)	317 (62.0)		
Ki67 group, *n* (%)					**0.015**	0.448
<15%	195 (18.8)	30 (12.8)	54 (18.7)	111 (21.7)		
15%–30%	297 (28.7)	71 (30.2)	88 (30.4)	138 (27.0)		
>30%	543 (52.5)	134 (57.0)	147 (50.9)	262 (51.3)		
Molecular subtype, *n* (%)					0.122	**0.017**
HR + HER2−	529 (51.1)	123 (52.3)	143 (49.5)	263 (51.5)		
HR + HER2+	113 (10.9)	19 (8.1)	47 (16.3)	47 (9.2)		
HR‐HER2+	71 (6.9)	8 (3.4)	24 (8.3)	39 (7.6)		
TNBC	322 (31.1)	85 (36.2)	75 (25.9)	162 (31.7)		
Tumor site, *n* (%)					0.189	0.628
≤2 cm	460 (44.5)	90 (38.3)	138 (47.7)	232 (45.4)		
>2 cm	528 (51.0)	132 (56.2)	141 (48.8)	255 (49.9)		
Unknown	47 (4.5)	13 (5.5)	10 (3.5)	24 (4.7)		
Lymph nodes status, *n* (%)					0.380	0.999
Negative	464 (44.8)	96 (40.9)	132 (45.7)	236 (46.2)		
Positive	541 (52.3)	131 (55.7)	149 (51.6)	261 (51.1)		
Unknown	30 (2.9)	8 (3.4)	8 (2.8)	14 (2.7)		
Metastasis, *n* (%)					0.346	0.852
Negative	971 (93.8)	224 (95.3)	272 (94.1)	475 (93.0)		
Positive	48 (4.6)	7 (3.0)	13 (4.5)	28 (5.5)		
Unknown	16 (1.6)	4 (1.7)	4 (1.4)	8 (1.6)		
Family history, *n* (%)					**<0.001**	0.352
BC	201 (19.4)	73 (31.1)	48 (16.6)	80 (15.7)		
Other *BRCA*‐related cancer	37 (3.6)	22 (9.4)	4 (1.4)	11 (2.1)		
Non‐*BRCA*‐related cancer	146 (14.1)	25 (10.6)	36 (12.5)	85 (16.6)		
No	651 (62.9)	115 (48.9)	201 (69.6)	335 (65.6)		
Other primary tumor, *n* (%)					**<0.001**	0.555
*BRCA*‐related cancer^a^	24 (2.3)	15 (6.4)	3 (1.0)	6 (1.2)		
Non‐*BRCA*‐related cancer	50 (4.8)	14 (6.0)	16 (5.5)	20 (3.9)		
No	961 (92.9)	206 (87.6)	270 (93.4)	485 (94.9)		
Bilateral BC, *n* (%)					**0.003**	0.495
Yes	85 (8.2)	33 (14.0)	16 (5.5)	36 (7.0)		
No	950 (91.8)	202 (86.0)	273 (94.5)	475 (93.0)		
NCCN high‐risk criteria					**<0.001**	0.901
Yes	906 (87.5)	222 (94.5)	246 (85.1)	438 (85.7)		
No	129 (12.5)	13 (5.5)	43 (14.9)	73 (14.3)		

Abbreviations: BC, breast cancer; DCIS, ductal carcinoma in situ; G, grade; HER2, human epidermal growth factor receptor 2; HR, hormone receptor; IDC, invasive ductal carcinoma; ILC, invasive lobular carcinoma; NCCN, National Comprehensive Cancer Network; OC, ovarian cancer; PaC, pancreatic cancer; PrC, prostate cancer; P/LPV, pathogenic or likely pathogenic variant; *P*_*1*_: P/LPV versus non‐variant; *P*_*2*_: VUS versus non‐variant; TNBC, triple negative breast cancer; VUS, variant of uncertain significance.

^a^ OC, PaC, and PrC.

^b^ Mucinous carcinoma, invasive micropapillary carcinoma, metaplastic carcinoma, and squamous cell carcinoma.

Bold *P* values indicate significant differences between the two variables.

### 3.2 Germline variant patterns in BC susceptibility genes

**FIGURE 2:**
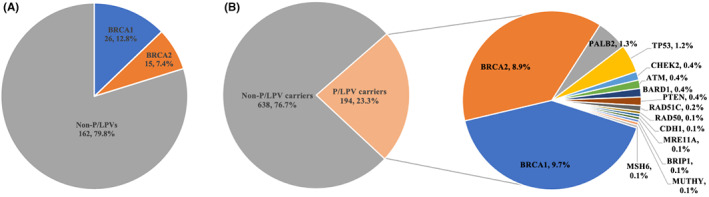
The distribution and frequency of germline pathogenic or likely pathogenic variants (P/LPVs). (A) 41 patients carried 41 P/LPVs in *BRCA1/2* testing. (B) 194 patients carried 196 P/LPVs in 21 genes panel testing.

The distribution and frequency of mutational genes were shown in Figure 2A,B. Among the study population, 203 (19.6%) patients tested only for *BRCA1/2*, and 832 (80.4%) received 21 genes panel testing. Among patients who had *BRCA1/2* testing alone, 41 (20.2%) were found to carry 41 P/LPVs, including 26 (12.8%) *BRCA1* and 15 (7.4%) *BRCA2* variants. Among patients who had panel testing, 194 (23.3%) were found to carry 196 P/LPVs, including 81 (9.7%) had *BRCA1* variants, 74 (8.9%) had *BRCA2* variants, 41 (4.9%) had non‐*BRCA* variants, and 2 patients carried two P/LPVs simultaneously (Table S2). The most common non‐*BRCA* P/LPVs were *PALB2* (11, 1.3%), *TP53* (10, 1.2%), *PTEN* (3, 0.4%), *CHEK2* (3, 0.4%), *ATM* (3, 0.4%), *BARD1* (3, 0.4%), and *RAD51C* (2, 0.2%). Eight *BRCA1* recurrent variants were observed, including c.5470_5477del (*n* = 10), c.3214del (*n* = 9), c.4065_4068del (*n* = 5), c.3472G > T (*n* = 3), c.1012A > T (*n* = 3), c.1898del (*n* = 3), c.5335del (*n* = 3), and c.4801A > T (*n* = 3) (Table S3). Seven *BRCA2* recurrent variants were found, including c.3109C > T (*n* = 6), c.67 + 2 T > A (*n* = 5), c.2806_2809del (*n* = 5), c.5722_5723del (*n* = 4), c.5164_5165del (*n* = 3), c.5645C > A (*n* = 3), and c.1454delinsTGTATT (*n* = 3) (Table S4). Among these recurrent variants, *BRCA1* c .5470_5477del and *BRCA2* c.3109C > T were two Chinese founder variants. Other Chinese founder variants such as *BRCA1* c.981_982del (*n* = 2) and *BRCA2* c.9097dup (*n* = 2) were also found in the study. *BRCA2* c.2806_2809del had previously been identified as a Colombian founder variant, and *BRCA2* c.5164_5165del had been identified as a potential founder variant in the Taiwanese population. No recurrent variant in non‐*BRCA* variants was observed, but one *PALB2* c.2257C > T truncating variant was a Greek founder, and one *PALB2* c.1592del had identified as a founder variant in the Finland population (Table S5). Notably, a number of novel germline P/LPVs which has not reported in public databases (ClinVar, LOVD) and in the literature before were found and highlighted in the Tables S3–S5, including seven *BRCA1* variants, eighteen *BRCA2* variants, and nine non‐*BRCA* variants. Rare P/LPVs were also highlighted.

**FIGURE 3:**
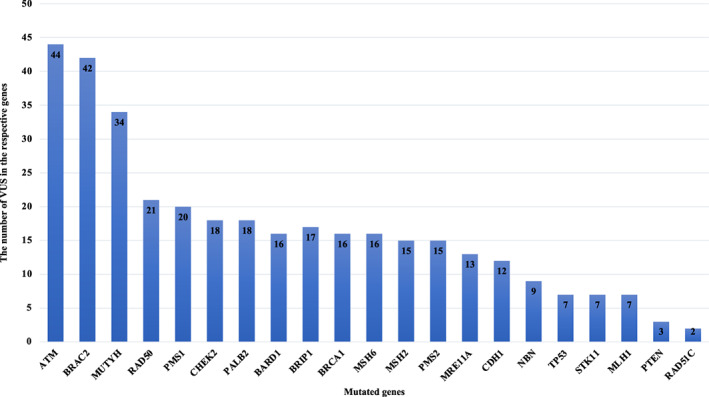
The frequency of variants of uncertain significance (VUSs).

In addition, 352 VUSs were detected in 289 patients, including 7 (3.4%) patients who had *BRCA1/2* testing alone and 282 (33.9%) had panel testing. The most common genes had VUSs were *ATM* (*n* = 44), *BRCA2* (*n* = 42), and *MUTYH* (*n* = 34) (Figure 3). All VUSs were summarized in Table S6.

### 3.3 Clinicopathological characteristics of P/LPV carriers

Compared with patients without variants, P/LPVs showed significant younger age at diagnosis (*p* = 0.004), higher rate of IDC (*p* = 0.029), higher histological grade (*p* = 0.014), high Ki67 index (*p* = 0.015), higher rate of family history of *BRCA*‐related cancer (*p* < 0.001), other primary *BRCA*‐related cancer (*p* < 0.001), and bilateral BC (*p* = 0.003) (Table 1). In addition, we found patients with *BRCA1*, *BRAC2*, and non‐*BRCA* P/LPVs showed significantly different clinicopathological characteristics. Compared with *BRCA1* P/LPVs, patients with non‐*BRCA* P/LPVs showed a lower percent of IDC subtype (*p* = 0.036) and grade 3 tumor (*p* = 0.015), lower Ki67 index (*p* = 0.002), lower percent of HR‐negative disease (*p* < 0.001), and TNBC subtype (*p* < 0.001), less family history of BC and other *BRCA*‐related cancers (*p* < 0.001), and less second primary *BRCA*‐related cancer (*p* = 0.011). Non‐*BRCA* P/LPV carriers showed a higher percentage of HER2 positive disease compared with BRCA1/2 variants, although some difference did not reach statistical significance (Table 2).

**TABLE 2:** The characteristics of germline pathogenic or likely pathogenic variants.

Variables	g*BRCA1* variant carriers (*N* = 107)	g*BRCA2* variant carriers (*N* = 88)	Non‐*BRCA* variant carriers (*N* = 40)	*P*_1_	*P*_2_
Gender, *n* (%)				0.272	>0.999
Male	0 (0)	3 (3.4)	1 (2.5)		
Female	107 (100)	85 (96.6)	39 (97.5)		
Age at diagnosis, Median (range)	39 (24, 68)	41 (26, 78)	37.5 (24, 68)	0.591	0.499
Age group, *n* (%)				0.422	0.079
≤30 y	13 (12.2)	11 (12.5)	2 (5.0)		
31–40 y	41 (38.3)	27 (30.7)	22 (55.0)		
41–50 y	30 (28.0)	32 (36.4)	8 (20.0)		
51–60 y	18 (16.8)	14 (15.9)	6 (15.0)		
≥60 y	5 (4.7)	4 (4.5)	2 (5.0)		
Histological subtype, *n* (%)				**0.036**	0.508
IDC	106 (99.1)	84 (95.5)	37 (92.5)		
DCIS	1 (0.9)	1 (1.1)	0 (0)		
ILC	0 (0)	3 (3.4)	2 (5.0)		
Special type^b^	0 (0)	0 (0)	1 (2.5)		
Histological grade, *n* (%)				**0.015**	0.326
G1 + G2	29 (27.1)	48 (54.6)	17 (42.5)		
G3	77 (72.0)	37 (42.0)	20 (50.0)		
Unknown	1 (0.9)	3 (3.4)	3 (7.5)		
HR status, *n* (%)				**<0.001**	0.185
Negative	63 (58.9)	12 (13.6)	10 (25.0)		
Positive	44 (41.1)	76 (86.4)	30 (75.0)		
Ki67 group, *n* (%)				**0.002**	0.908
<15%	6 (5.6)	17 (19.3)	7 (17.5)		
15–30%	21 (19.6)	35 (39.8)	15 (37.5)		
>30%	80 (74.8)	36 (40.9)	18 (45.0)		
Molecular subtype, *n* (%)				**<0.001**	0.061
HR + HER2‐	37 (34.6)	64 (72.7)	22 (55.0)		
HR + HER2+	2 (1.9)	10 (11.4)	7 (17.5)		
HR‐HER2+	1 (0.9)	2 (2.3)	5 (12.5)		
TNBC	67 (62.6)	12 (13.6)	6 (15.0)		
Tumor site, *n* (%)				0.094	**0.002**
≤2 cm	42 (39.3)	28 (31.8)	20 (50.0)		
>2 cm	59 (55.1)	58 (65.9)	15 (37.5)		
Unknown	6 (5.6)	2 (2.3)	5 (12.5)		
Lymph nodes status, *n* (%)				0.596	0.116
Negative	47 (43.9)	33 (37.5)	16 (40.0)		
Positive	56 (52.3)	54 (61.4)	21 (52.5)		
Unknown	4 (3.7)	1 (1.1)	3 (7.5)		
Metastasis, *n* (%)				0.552	0.519
Negative	103 (96.3)	84 (95.5)	37 (92.5)		
Positive	2 (1.9)	3 (3.4)	2 (5.0)		
Unknown	2 (1.9)	1 (1.1)	1 (2.5)		
Family history, *n* (%)				**<0.001**	0.153
BC	40 (37.4)	28 (31.8)	5 (12.5)		
Other *BRCA*‐related cancer	15 (14.0)	5 (5.7)	2 (5.0)		
Non‐*BRCA*‐related cancer	13 (12.1)	7 (8.0)	4 (10.0)		
No	39 (36.5)	48 (54.5)	29 (72.5)		
Other primary tumor, *n* (%)				**0.011**	**0.017**
*BRCA*‐related cancer^a^	10 (9.3)	5 (5.7)	0 (0)		
Non‐*BRCA*‐related cancer	5 (4.7)	3 (3.4)	6 (15.0)		
No	92 (86.0)	80 (90.9)	34 (85.0)		
Bilateral BC, *n* (%)				0.627	0.219
Yes	16 (15.0)	9 (10.2)	8 (20.0)		
No	91 (85.0)	79 (89.8)	32 (80.0)		
NCCN high‐risk criteria				0.398	>0.999
Yes	104 (97.2)	81 (92.0)	37 (92.5)		
No	3 (2.8)	7 (8.0)	3 (7.5)		

Abbreviations: BC, breast cancer; DCIS, ductal carcinoma in situ; G, grade; HER2, human epidermal growth factor receptor 2; HR, hormone receptor; IDC, invasive ductal carcinoma; ILC, invasive lobular carcinoma; NCCN, National Comprehensive Cancer Network; OC, ovarian cancer; P/LPV, pathogenic or likely pathogenic variant; *P*_*1*_: g*BRCA1* versus Non‐*BRCA*; *P*_*2*_: g*BRCA2* versus Non‐*BRCA*; PaC, pancreatic cancer; PrC, prostate cancer; TNBC, triple negative breast cancer; VUS, variant of uncertain significance.

^a^ OC, Pac, and Prc.

^b^ Mucinous carcinoma, invasive micropapillary carcinoma, metaplastic carcinoma, and squamous cell carcinoma.

Bold *P* values indicate significant differences between the two variables.

When we stratified the cohort according to molecular subtypes based on ER, PR, and HER2 expression, we found that the TNBC group had the highest P/LPV rate (26.4%), followed by HR + HER2‐ group (23.3%), HR + HER2+ group (16.8%), and HR‐HER2+ group (11.3%) (Table 3). Moreover, different molecular subgroups showed different variant profiles. The TNBC group had the highest incidence of *BRCA1* variants (20.8%). HR + HER2‐ BC patients showed the highest rate of *BRCA2* variants (12.3%), followed by *BRCA1* (7.0%) and *PALB2* variants (1.5%). HER2‐positive patients showed a higher rate of non‐*BRCA* variants than other groups, especially *TP53*, representing 3.5% (4/113) in the HR + HER2+ group and 4.2% (3/71) in the HR‐HER2+ group.

**TABLE 3:** P/LPV distribution in different molecular subgroups based on HR and HER2 status.

Cancer susceptibility genes	Molecular subgroups			
	HR + HER2− (*N* = 529)	HR + HER2+ (*N* = 113)	HR‐HER2+ (*N* = 71)	HR‐HER2− (*N* = 322)
P/LPV, *n* (%)	123 (23.3)	19 (16.8)	8 (11.3)	85 (26.4)
*BRCA1*, *n* (%)	37^a^ (7.0)	2 (1.8)	1 (1.4)	67^b^ (20.8)
*BRCA2*, *n* (%)	65^a^ (12.3)	10 (8.8)	2 (2.8)	12 (3.7)
Non‐*BRCA*, *n* (%)	22 (4.2)	7 (6.2)	5 (7.0)	7 (2.2)
*PALB2*, *n* (%)	8 (1.5)	1 (0.9)	1 (1.4)	1 (0.3)
*TP53*, *n* (%)	2 (0.4)	4 (3.5)	3 (4.2)	1 (0.3)
*CDH1*, *n* (%)	1 (0.2)	0	0	0
*ATM*, *n* (%)	2 (0.4)	0	0	1 (0.3)
*PTEN*, *n* (%)	2 (0.4)	0	0	1 (0.3)
*CHEK2*, *n* (%)	3 (0.6)	0	0	0
*BARD1*, *n* (%)	2 (0.4)	1 (0.9)	0	0
*RAD51C*, *n* (%)	0	0	1 (1.4)	1 (0.3)
*RAD50*, *n* (%)	0	0	0	1 (0.3)
*MRE11A*, *n* (%)	1 (0.2)	0	0	0
*MSH6*, *n* (%)	0	0	0	1^b^ (0.3)
*MUTYH*, *n* (%)	0	1 (0.9)	0	0
*BRIP1*, *n* (%)	1 (0.2)	0	0	0

^a^ A HR+HER2− patient carried both *BRCA1* and *BRCA2* variants.

^b^ A HR−HER2− patient carried both *BRCA1* and *MSH6* variants.

### 3.4 Comparison of different criteria for HBC

We applied different screening criteria available to our population cohort and compared the number of patients who met the criteria and had P/LPV (Table 4). At last, we found using Desai's criteria of testing all females diagnosed with BC by the age of 60 years and NCCN criteria for older patients could find most P/LPVs by testing the least number of patients.

**TABLE 4:** Comparison of different criteria for hereditary breast cancer testing.

Different criteria	Total, *n* (%)	P/LPVs, *n* (%)	Non‐P/LPVs, *n* (%)
NCCN criteria			
Yes	906 (87.5)	222 (94.5)	684 (85.5)
No	129 (12.5)	13 (5.5)	116 (14.5)
Yadav's hybrid criteria^c^			
Yes	1025 (99.0)	234 (99.6)	791 (98.9)
No	10 (1.0)	1 (0.4)	9 (1.1)
Desai's modified hybrid criteria^a^			
Yes	1011 (97.7)	234 (99.6)	777 (97.1)
No	24 (2.3)	1 (0.4)	23 (2.9)
Boddicker's criteria^b^			
Yes	915 (88.4)	222 (94.5)	693 (86.6)
No	120 (11.6)	13 (5.5)	107 (13.4)
Desai's modified hybrid criteria + all TNBC			
Yes	1020 (98.6)	234 (99.6)	786 (98.3)
No	15 (1.4)	1 (0.4)	14 (1.7)

^a^ Based on an additional comprehensive analysis of Mayo Clinic data: universal testing for all BC patients diagnosed by age 60 years (rather than by age 65) and use family‐based criteria, such as NCCN high‐risk criteria, for patients diagnosed after the age of 60 years.

^b^ Other authors have proposed: testing all triple‐negative breast cancers and the NCCN high‐risk criteria for other patients.

^c^ A novel hybrid approach proposed by Yadav et al: testing all breast cancer (BC) patients diagnosed by age 65 years and using NCCN high‐risk criteria for older patients.

## 4 DISCUSSION

The current study demonstrated the genetic and clinicopathological characteristics in a large cohort of Chinese HBC patients. Among the screened 1035 patients, 906 (87.5%) met the NCCN high‐risk criteria, and 235 were identified to carry at least one P/LPV in 15 BC susceptibility genes, with an overall P/LPVs rate of 22.7%, and 24.5% in the high‐risk population. Similar frequencies of P/LPVs have been reported in other studies of the Chinese population with high‐risk characteristics.^22^, ^23^ Even though, P/LPVs were detected for 13 out of 129 (10.1%) patients who did not meet the NCCN criteria for testing, suggesting NCCN criteria will miss a significant number of patients with HBC in the Chinese population.

The high missing rate of NCCN criteria has been demonstrated in studies of other ethnicities.^8^, ^24^ This is mainly due to the restrictive criteria for testing. On the other hand, universal genetic testing will create many other challenges such as high costs and genetic testing burdens. Given the limitations of these two genetic testing strategies, several other criteria have been proposed. The main difference among these criteria focused on the appropriate screening age. The Mayo Clinic hybrid approach by Yadav et al. reported that testing all female BC by the age of 65 years and using NCCN criteria for older patients could miss fewer variants than with NCCN criteria alone and spared 21% of patients for testing compared with universal screening.^24^ A subsequent report by Desai et al. demonstrated that lowering the age from 65 to 60 years maintained the detection sensitivity of Yadav's criteria >90% while sparing testing for an additional 10% of the population.^25^ Boddicker et al. showed that the frequency of *BRCA1/2* or *PALB2* variants in TNBC older than 65 years was 3.0%, thus supporting genetic testing for TNBC at any age.^26^ When we applied these criteria to our patients, we found using Desai's criteria of testing all females diagnosed with BC by the age of 60 years and NCCN criteria for older patients could find most P/LPVs by testing the least number of patients. However, due to the highly selective patients in the current study, further studies enrolling larger‐scale consecutive populations are needed to establish the appropriate criteria for Chinses patients.

Nowadays, multigene panel testing is increasingly used for HBC screening.^14^ In our cohort, patients who were diagnosed before 2017 received *BRCA1/2* testing alone, after then most patients underwent the 21 genes panel testing. Compared with *BRCA1/2* testing alone, panel testing identified 4.9% of non‐*BRCA* P/LPVs and a significantly high rate of VUSs (33.9%). The incidence of non‐*BRCA* P/LPVs varied dramatically in different studies, ranging from 1% to 12%. Moreover, an even large difference of VUSs was reported, ranging from 0.6 to 88%.^14^, ^27^, ^28^ The main reason probably due to the diversity of gene panels applied in different studies. However, more genes do not mean better. Many genes included in the panels are low‐risk or even unrelated to BC. Therefore, it will lead to the detection of more P/LPVs and VUSs without clinical significance.^29^ Besides *BRCA1/2*, 19 non‐*BRCA* genes were included in our panel. And 13 non‐*BRCA* variants were found, including *PALB2* (11, 1.3%), *TP53* (10, 1.2%), *PTEN* (3, 0.4%), *CHEK2* (3, 0.4%), *ATM* (3, 0.4%), *BARD1* (3, 0.4%), *RAD51C* (2, 0.2%), and *RAD50*, *CDH1*, *MRE11A*, *BRIP1*, *MSH6*, *MUTYH* (1, 0.1%). *PALB2* is the most prominent non‐*BRCA* gene, and testing *PALB2* is cost‐effective.^15^ *PALB2* works as the partner and localizer of *BRCA2*, and associates with an overall increased risk of BC of five to nine‐fold.^30^ Two large cohort studies demonstrated a greater association between *PALB2* P/LPVs and ER‐negative BC, whereas our data found *PALB2* variant was more common in HR‐positive patients, accounting for 9 of 11 cases.^26^, ^30^ Several studies have reported very low frequencies of germline *TP53* variants in the general BC population: 0.15% in Couch's study involving 41,603 Caucasian BC, 0.3% in Rarn's study involving 44,086 non‐selected population,^5^, ^31^ and 0.5% in another study containing 10,053 non‐selected Chinese BC patients.^32^ While a higher frequency has been reported in a high‐risk population.^33^ According to our results, *TP53* is one of the most common non‐*BCRA* variants, supporting the inclusion of *TP53* in genetic testing for the Chinese population. The association between MMR genes and the risk of BC remains controversial.^34^, ^35^ In the current study, we found one patient carrying *MSH6* and *BRCA1* PVs simultaneously and without any other personal or family history of cancer, while all the other MMR variants identified were VUSs. Since Wu B et al. reported two *MSH2* variants used to classify VUS are pathogenic mutations.^36^ Therefore, these VUS should be investigated further in a large number of patients. A case of *CDH1* PV was found in one female patient diagnosed with ILC at 40 years. She had a strong family history of gastric cancer, including her grandmother, father, and aunt.

Notably, we found non‐*BRCA* P/LPVs showed a low incidence of NCCN criteria listed family history of BC, OC, PaC, and PrC, as well as a low frequency of secondary primary cancer of OC, PaC, and PrC, suggesting testing criteria for non‐*BRCA* or panel testing should be not only based on these personal and family histories. In addition, different molecular subtypes exhibited different variant profiles. Consistent with other reports, TNBC had the highest proportion of *BRCA1* variants, and HR + HER2‐ BC had higher rates of *BRAC2* and *PALB2* variants. While HER2‐positive BC patients had the highest non‐*BRCA* P/LPVs, especially *TP5* variants. Therefore, future criteria probably should take into consideration of molecular factors, but not just focus on HER2‐negative or TNBC patients.

The main limitation of our current study is that it enrolled the highly selective high‐risk patients. Larger scale studies in consecutive populations should be initiated to further explore optimal genetic testing strategies for BC.

## 5 CONCLUSION

Our current study suggested Desai's criteria of testing all females diagnosed with BC by the age of 60 years and using NCCN criteria for older patients, might be a more suitable genetic testing criteria for Chinese BC patients. Panel testing could identify more non‐*BRCA* P/LPVs than *BRCA1/2* testing alone. Non‐*BRCA* P/LPVs showed different personal and family histories of cancer and molecular subtype distributions compared with *BRCA1/2* P/LPVs. The optimal genetic testing strategy for BC still needs to be investigated by larger‐scale consecutive population studies.

## AUTHOR CONTRIBUTIONS

**Meng‐qian Ni:** Data curation (lead); formal analysis (lead); visualization (lead); writing – original draft (lead). **Fang Wang:** Data curation (lead); writing – original draft (equal). **Anli Yang:** Supervision (equal); writing – review and editing (equal). **Qiong Shao:** Writing – original draft (equal). **Cong Xue:** Data curation (equal). **Wen Xia:** Data curation (equal). **Fei Xu:** Data curation (equal). **Xi Lin:** Data curation (equal). **Jia‐Jia Huang:** Data curation (equal). **Xiwen Bi:** Data curation (equal). **Ruoxi Hong:** Data curation (equal). **Meiting Chen:** Data curation (supporting). **Qiufan Zheng:** Data curation (equal). **Kuikui Jiang:** Data curation (equal). **Xinhua Xie:** Data curation (equal). **Jun Tang:** Data curation (equal). **Xi Wang:** Data curation (equal). **Zhong‐yu Yuan:** Data curation (equal). **Shusen Wang:** Conceptualization (equal); data curation (equal); supervision (equal). **Yanxia Shi:** Conceptualization (equal); funding acquisition (lead); supervision (equal). **Xin An:** Project administration (equal); writing – review and editing (equal).

## FUNDING INFORMATION

This work was supported by the National Key Research and Development Program (2021YFE0206300), National Natural Science Foundation of China (81773279, 82073391 to Dr. Yanxia Shi), Science and Technology Planning Project of Guangdong Province (2016A050502015, 2013B021800062 and 2012B061700082 to Dr. Yanxia Shi).

## CONFLICT OF INTEREST STATEMENT

No potential conflicts of interest were disclosed.

## Supporting information


Tables S1–S6.

## Data Availability

The datasets generated for this study are available on request to the corresponding author.
