# Supplementary material for: What is the appropriate genetic testing criteria for breast cancer in the Chinese population?—Analysis of genetic and clinical features from a single cancer center database
Source: Cancer Med. 2023 Apr 25;12(12):13019–30. doi: 10.1002/cam4.5976 (PMC10315755; doi:10.1002/cam4.5976)
Supplement: Supplementary file 1 — Tables S1–S6. [file CAM4-12-13019-s001.docx]

**Supplementary table 1.** **Stratification of 21 genes panel testing.**

| **Name of genes** | **Gene transcript** | **Function of genes** |
| --- | --- | --- |
| *BRCA1* | NM_007294 | BRCA1-associated protein complex |
| *BARD1* | NM_000465 |  |
| *STK11* | NM_999455 | Cell cycle regulation |
| *CDH1* | NM_004360 | Cell adhesion |
| *TP53* | NM_000546 | Cell growth |
| *PTEN* | NM_000314 | PI3K/MAPK signaling pathway |
| *BRCA2* | NM_000059 | Fanconi/BRCA |
| *PALB2* | NM_024675 |  |
| *RAD51C* | NM_058216 |  |
| *BRIP1* | NM_032043 |  |
| *CHEK2* | NM_007194 | DNA repair |
| *ATM* | NM_000051 |  |
| *RAD50* | NM_005731 |  |
| *MUTYH* | NM_001128425 |  |
| *MRE11A* | NM_005590 |  |
| *NBN* | NM_002485 |  |
| *MLH1* | NM_000249 | Mismatch repair |
| *MSH2* | NM_000251 |  |
| *MSH6* | NM_000179 |  |
| *PMS1* | NM_000534 |  |
| *PMS2* | NM_000535 |  |

**Supplementary table 2. Patients with more than one variant.**

| **Patient ID** | **Nucleotide and amino acid change** | | **Variant type** | | **Diagnosis age** | **Molecular subgroup** | **Family history of cancer** |
| --- | --- | --- | --- | --- | --- | --- | --- |
| 419237 | *BRCA1* c.3214del; p.Glu1701_Leu1072insTer  *BRCA2* c.7816dup; p.Asp2606fs |  | | Frameshift  Frameshift | 29 | HR+HER2- | None |
| 489149 | *BRCA1* c.3214del; p.Glu1701_Leu1072insTer  *MSH6* c.2230dup; p.Glu744fs |  |  | Frameshift  Frameshift | 31 | TNBC | None |

**Supplementary table 3. Distribution of *BRCA1*# P/LPVs in 107 patients.**

| **Patient ID** | **Nucleotide and amino acid change** | **Variant type** | **Variant status** | **Diagnosis age (years)** | **Molecular subgroup** | **Secondary BRCA-related cancer** | **Family history of cancer** |
| --- | --- | --- | --- | --- | --- | --- | --- |
| 344165 | exon2:c.34C>T:p.Gln12Ter | Nonsense |  | 43 | TNBC | None | None |
| M2673723 | exon2:c.65T>G:p.Leu22Ter | Nonsense | Rare | 32 | TNBC | None | None |
| 323801 | exon2:c.66_67insA:p.Glu23fs | Frameshift |  | 50 | TNBC | None | Mother: lung cancer |
| 544367 | exon2:c.66dup:p.Glu23fs | Frameshift |  | 43 | TNBC | None | Mother: BC |
| 469701 | exon2:c.80+5G>A | Splice site | Rare | 51 | HR+HER2- | None | Mother: BC; father: liver cancer |
| 435257 | exon3:c.134+1G>C | Splice site |  | 38 | TNBC | None | Father: lung cancer |
| M1696837 | exon4:c.212G>A:p.Arg71Lys | Missense |  | 51 | TNBC | None | Sister: OC*1 |
| M2486850 | exon4:c.212G>A:p.Arg71Lys | Missense |  | 33 | TNBC | None | None |
| 493963 | exon4:c.283_286del:p.Leu95fs | Frameshift | Rare | 32 | HR+HER2- | None | Mother: BC |
| 380919 | exon5:c.302-1G>A | Splice site |  | 38 | HR+HER2+ | None | None |
| 527169 | exon6:c.438del:p.Leu147fs | Frameshift | Novel | 48 | HR+HER2- | None | Mother: OC |
| 109460 | exon7:c.470_471del:p.Leu156_Ser157insTer | Frameshift |  | 62 | TNBC | OC | Father: lung cancer |
| 448667 | exon10:c.981_982del:p.Thr327_Cys328insTer | Frameshift |  | 46 | HR+HER2- | None | Mother and aunt: BC*2; father: lung cancer and renal carcinoma |
| 534798 | exon10:c.981_982del:p.Thr327_Cys328insTer | Frameshift |  | 35 | TNBC | None | Mother: OC |
| 416012 | exon10:c.1012A>T:p.Lys338Ter | Nonsense | Recurrent | 68 | TNBC | None | Aunt: BC*1 |
| 387603 | exon10:c.1012A>T:p.Lys338Ter | Nonsense | Recurrent | 53 | HR+HER2- | None | None |
| 400530 | exon10:c.1012A>T:p.Lys338Ter | Nonsense | Recurrent | 33 | HR+HER2- | None | None |
| 532669 | exon10:c.1115G>A:p.Trp372Ter | Nonsense |  | 48 | TNBC | None | Father: lung cancer |
| 553961 | exon10:c.1214C>G:p.Ser405Ter | Nonsense |  | 42 | TNBC | None | Mother and sister: BC*2 |
| 346085 | exon10:c.1480C>T:p.Gln494Ter | Nonsense |  | 60 | HR+HER2- | None | Sister and niece: BC*3 |
| 292119 | exon10:c.1504_1508del:p.Leu502fs | Frameshift |  | 33 | TNBC | None | None |
| 307622 | exon10:c.1898del:p.Pro633fs | Frameshift | Recurrent | 47 | TNBC | OC | Sister: BC*1 |
| 426306 | exon10:c.1898del:p.Pro633fs | Frameshift | Recurrent | 45 | HR+HER2- | None | None |
| 410117 | exon10:c.1898del:p.Pro633fs | Frameshift | Recurrent | 43 | TNBC | None | Aunt: OC*1 |
| 433777 | exon10:c.1961dup:p.Tyr655fs | Frameshift |  | 53 | HR+HER2- | None | Father: stomach cancer |
| 382369 | exon10:c.2217_2218insAA:p.Val740fs | Frameshift | Novel | 30 | TNBC | None | None |
| 460465 | exon10:c.2217_2218insAA:p.Val740fs | Frameshift | Novel | 35 | TNBC | None | Aunt: BC*1 |
| 439668 | exon10:c.2253_2254del:p.Met751fs | Frameshift |  | 36 | HR+HER2- | None | None |
| 413872 | exon10:c.2269del:p.Val757fs | Frameshift |  | 39 | TNBC | None | Mother and sister: BC*2 |
| 423230 | exon10:c.2273T>A:p.Leu758Ter | Nonsense |  | 60 | TNBC | None | Mother: BC |
| 481774 | exon10:c.2273T>A:p.Leu758Ter | Nonsense |  | 53 | HR+HER2- | None | Sister: BC*1 |
| M2446670 | exon10:c.2545G>T:p.Glu849Ter | Nonsense |  | 59 | HR+HER2- | OC | Brother: PrC*1; father: stomach cancer |
| 285775 | exon10:c.2545G>T:p.Glu849Ter | Nonsense |  | 52 | HR+HER2- | PaC | None |
| M2205638 | exon10:c.2572C>T:p.Gln858Ter | Nonsense |  | 30 | TNBC | None | None |
| 161182 | exon10:c.2572C>T:p.Gln858Ter | Nonsense |  | 26 | TNBC | None | Mother: BC and OC; aunt: BC*1; grandfather: stomach cancer*1 |
| M2194268 | exon10:c.2611_2612del:p.Pro871fs | Frameshift |  | 47 | TNBC | None | Sister: BC*1 and OC*1; mother: colon cancer; father: lung cancer |
| 374801 | exon10:c.2635G>T:p.Glu879Ter | Nonsense |  | 39 | TNBC | None | None |
| 335308 | exon10:c.2635G>T:p.Glu879Ter | Nonsense |  | 34 | HR+HER2- | None | None |
| 484382 | exon10:c.2643dup:p.Cys882fs | Frameshift | Rare | 28 | HR+HER2- | None | None |
| 401132 | exon10:c.2654del:p.Phe885fs | Frameshift |  | 46 | HR+HER2- | None | Brother: lung cancer*1 |
| 149661 | exon10:c.3181del:p.Glu1060_Ile1061insTer | Nonsense |  | 51 | HR+HER2- | OC | None |
| 468806 | exon10:c.3214del:p.Glu1701_Leu1072insTer | Nonsense | Recurrent | 59 | HR+HER2- | OC | Mother: OC; father: PrC |
| 260505 | exon10:c.3214del:p.Glu1701_Leu1072insTer | Frameshift | Recurrent | 46 | TNBC | OC | Aunt: BC*1; father: bladder Cancer; brother: rectal cancer*1 |
| 511029 | exon10:c.3214del:p.Glu1701_Leu1072insTer | Frameshift | Recurrent | 39 | HR+HER2- | None | Mother: OC |
| 472809 | exon10:c.3214del:p.Glu1701_Leu1072insTer | Frameshift | Recurrent | 38 | HR+HER2+ | None | Father: stomach cancer |
| 423229 | exon10:c.3214del:p.Glu1701_Leu1072insTer | Frameshift | Recurrent | 36 | TNBC | None | None |
| 461449 | exon10:c.3214del:p.Glu1701_Leu1072insTer | Frameshift | Recurrent | 32 | TNBC | None | None |
| 489149 | exon10:c.3214del:p.Glu1701_Leu1072insTer | Frameshift | Recurrent | 31 | TNBC | None | None |
| 419237 | exon10:c.3214del:p.Glu1701_Leu1072insTer | Frameshift | Recurrent | 29 | HR+HER2- | None | None |
| 556471 | exon10:c.3214del:p.Glu1701_Leu1072insTer | Nonsense | Recurrent | 31 | HR+HER2- | None | Sister: BC*1; grandfather and uncle: lung cancer*2 |
| 165110 | exon10:c.3301del:p.Ser1101fs | Frameshift | Novel | 43 | HR+HER2- | OC | Aunt: BC*1; sister: OC*2 |
| 465155 | exon10:c.3424del:p.ALa1142fs | Frameshift |  | 36 | TNBC | None | None |
| 184008 | exon10:c.3461T>G:p.Leu1154Ter | Nonsense |  | 60 | HR+HER2- | OC | None |
| 296340 | exon10:c.3472G>T:p.Glu1158Ter | Nonsense | Recurrent | 55 | TNBC | None | None |
| 279716 | exon10:c.3472G>T:p.Glu1158Ter | Frameshift | Recurrent | 41 | TNBC | None | None |
| 462072 | exon10:c.3472G>T:p.Glu1158Ter | Nonsense | Recurrent | 39 | TNBC | None | Aunt: BC*1 |
| 386450 | exon10:c.3607C>T:p.Arg1203Ter | Nonsense |  | 38 | HR+HER2- | None | None |
| 468349 | exon10:c.3653_3654insATAG:p.Ser1218fs | Frameshift | Novel | 38 | TNBC | None | Mother: colon cancer |
| 421491 | exon10:c.3770_3771del:p.Glu1257fs | Frameshift |  | 33 | TNBC | None | None |
| 428503 | exon10:c.3870_3871insA:p.Cys1291fs | Frameshift | Novel | 58 | TNBC | None | Mother: BC; sister: OC*1 |
| 495954 | exon10:c.3967_3970del:p.Lys1322_Gln1323insTer | Frameshift | Rare | 29 | TNBC | None | None |
| 482445 | exon10:c.4065_4068del:p.Asn1355fs | Frameshift | Recurrent | 57 | TNBC | None | Sister: OC*1 |
| 450849 | exon10:c.4065_4068del:p.Asn1355fs | Frameshift | Recurrent | 46 | TNBC | None | None |
| 442005 | exon10:c.4065_4068del:p.Asn1355fs | Frameshift | Recurrent | 43 | HR+HER2- | None | Mother: BC |
| 525700 | exon10:c.4065_4068del:p.Asn1355fs | Frameshift | Recurrent | 42 | TNBC | None | Aunt: OC*1 |
| 458093 | exon10:c.4065_4068del:p.Asn1355fs | Frameshift | Recurrent | 35 | TNBC | None | Sister: OC*1 |
| 438911 | exon10:c.4075C>T:p.Gln1359Ter | Nonsense |  | 33 | TNBC | None | None |
| 461168 | exon10:c.4075C>T:p.Gln1359Ter | Nonsense |  | 29 | HR+HER2- | None | Uncle: colon cancer*2 |
| 546226 | exon10:c.4097-1G>A | Splice site |  | 29 | TNBC | None | Mother: BC |
| 464779 | exon11:c.4148C>G:p.Ser1383Ter | Nonsense |  | 38 | TNBC | None | None |
| 282087 | exon11:c.4148C>G:p.Ser1383Ter | Nonsense |  | 32 | TNBC | None | None |
| 551899 | exon11:c.4185+1G>A | Splice site |  | 51 | TNBC | None | None |
| 472521 | exon11:c.4185+2T>G | Splice site | Rare | 51 | HR+HER2- | None | Aunt: BC*1 |
| 481440 | exon12:c.4335_4338dup:p.Gln1447fs | Frameshift |  | 39 | HR+HER2- | None | Mother: OC; sister: BC*1 |
| M2418806 | exon14:c.4573C>T:p.Gln1525Ter | Nonsense | Rare | 30 | TNBC | None | None |
| 486488 | exon15:c.4801A>T:p.Lys1601Ter | Nonsense | Recurrent | 37 | HR+HER2- | None | Aunt and sister: BC*2 |
| 470510 | exon15:c.4801A>T:p.Lys1601Ter | Nonsense | Recurrent | 35 | TNBC | None | Mother: OC |
| 328915 | exon15:c.4801A>T:p.Lys1601Ter | Nonsense | Recurrent | 35 | TNBC | None | Mother: BC |
| 466311 | exon15:c.4834C>T:p.Gln1612Ter | Nonsense |  | 34 | TNBC | None | Aunt: BC*2 |
| 433314 | exon15:c.4953_4954insC:p.Met1652fs | Frameshift | Novel | 31 | TNBC | None | None |
| 467297 | exon15:c.4986+4A>T | Splice site |  | 40 | TNBC | None | Mother: BC |
| 501033 | exon16:c.5074G>A:p.Asp1692Asn | Missense |  | 38 | HR+HER2- | None | Aunt and sister: BC*2 |
| 411581 | exon16:c.5074+3A>G | Splice site |  | 43 | TNBC | None | Aunt and sister: BC*3 |
| 484735 | exon19:c.5251C>T:p.Arg1751Ter | Nonsense |  | 43 | HR+HER2- | None | None |
| 484611 | exon21:c.5335del:p.Gln1779fs | Frameshift | Recurrent | 40 | TNBC | None | Grandmother: BC*1; father: lung cancer |
| 392147 | exon21:c.5335del:p.Gln1779fs | Frameshift | Recurrent | 25 | HR+HER2- | None | Grandmother: colon cancer |
| M2022596 | exon21:c.5335del:p.Gln1779fs | Frameshift | Recurrent | 24 | HR+HER2- | None | None |
| 212392 | exon21:c.5345G>A:p.Trp1782Ter | Nonsense |  | 42 | TNBC | None | Father: bladder cancer |
| 496627 | exon21:c.5387C>A:p.Ser1796Ter | Nonsense |  | 48 | TNBC | None | Sister: BC*2 |
| 469583 | exon22:c.(5406+1_5407-1)_(*1383_?) del | Deletion |  | 28 | TNBC | None | Sister: OC*1 |
| 407749 | exon23:c.5470_5477del:p.Ile1824fs | Frameshift | Recurrent | 40 | TNBC | None | None |
| 458793 | exon23:c.5470_5477del:p.Ile1824fs | Frameshift | Recurrent | 43 | TNBC | OC | Mother: PaC; aunt: BC*1 and colon*2; sister: BC*3; Uncle: liver cancer*1 |
| 402152 | exon23:c.5470_5477del:p.Ile1824fs | Frameshift | Recurrent | 42 | HR-HER2+ | None | Aunt: BC*2 |
| 556461 | exon23:c.5470_5477del:p.Ile1824fs | Frameshift | Recurrent | 56 | TNBC | None | Mother: OC; sister: BC*3 and BC*1; brother: liver cancer*1 |
| 448610 | exon23:c.5470_5477del:p.Ile1824fs | Frameshift | Recurrent | 50 | TNBC | None | Aunt and sister: BC*2 |
| 365378 | exon23:c.5470_5477del:p.Ile1824fs | Frameshift | Recurrent | 44 | HR+HER2- | None | None |
| 542237 | exon23:c.5470_5477del:p.Ile1824fs | Frameshift | Recurrent | 38 | TNBC | None | None |
| 434151 | exon23:c.5470_5477del:p.Ile1824fs | Frameshift | Recurrent | 37 | TNBC | None | Mother: BC |
| 329111 | exon23:c.5470_5477del:p.Ile1824fs | Frameshift | Recurrent | 35 | TNBC | None | Aunt: BC*1 |
| 452563 | exon23:c.5470_5477del:p.Ile1824fs | Frameshift | Recurrent | 34 | TNBC | None | Sister: BC*1 |
| 444041 | exon23:c.5511G>C:p.Trp1837Cys | Missense |  | 42 | TNBC | None | Mother: OC |
| 553949 | exon23:c.5521A>C:p.Ser1841Arg | Missense |  | 48 | TNBC | None | Father: colon cancer |
| 430624 | exon23:c.5521del:p.Ser1841fs | Frameshift |  | 42 | HR+HER2- | None | Mother and aunt: BC*2 |
| 523270 | exon1_13 del | Deletion |  | 31 | HR+HER2- | None | Mother and aunt: BC*2 |
| 376882 | exon1_2 del | Deletion |  | 52 | TNBC | None | Sister: OC*1 |
| M2664302 | exon8_9 del | Deletion |  | 36 | TNBC | None | None |
| 508755 | exon20 del | Deletion |  | 30 | HR+HER2- | None | Aunt: OC*1; grandfather: lung cancer*1 |

**#: *BRCA1* (NM_007294.3).** **Abbreviations: BC, breast cancer; OC, ovarian cancer; PaC, pancreatic cancer; PrC, prostate cancer.**

**Supplementary table 4.** **Distribution of *BRCA2*# P/LPVs in 88 patients.**

| **Patient ID** | **Nucleotide and amino acid change** | **Variant type** | **Variant status** | **Diagnosis age (years)** | **Molecular subgroup** | **Secondary BRCA-related cancer** | **Family history of cancer** |
| --- | --- | --- | --- | --- | --- | --- | --- |
| 420466 | exon2:c.67G>A:p.Asp23Asn | Missense | Rare | 46 | HR+HER2- | None | Mother and sister: BC*2 |
| 308366 | exon2:c.67+2T>A | Splice site | Rare/ Recurrent | 54 | HR+HER2- | None | None |
| 495421 | exon2:c.67+2T>A | Splice site | Rare/ Recurrent | 47 | HR+HER2- | None | None |
| 290654 | exon2:c.67+2T>A | Splice site | Rare/ Recurrent | 36 | HR+HER2- | None | None |
| 459427 | exon2:c.67+2T>A | Splice site | Rare/ Recurrent | 34 | HR+HER2- | None | None |
| 191595 | exon7:c.631G>A:p.Val11Ile | Missense |  | 62 | HR+HER2- | OC | None |
| 502326 | exon9:c.774_775del:p.Glu260fs | Frameshift |  | 39 | HR-HER2+ | None | None |
| 507702 | exon10:c.961C>T:p.Gln321Ter | Nonsense |  | 44 | TNBC | None | Father: PrC |
| M1910165 | exon10:c.961C>T:p.Gln321Ter | Nonsense |  | 43 | HR+HER2- | None | None |
| 358254 | exon10:c.1296_1297del:p.Asn433fs | Frameshift |  | 38 | HR+HER2- | None | None |
| 315775 | exon10:c.1454delinsTGTATT:p.Lys485fs | Frameshift | Rare/ Recurrent | 59 | HR+HER2- | OC | Aunt: lung cancer*1 |
| 421560 | exon10:c.1454delinsTGTATT:p.Lys485fs | Frameshift | Rare/ Recurrent | 36 | HR+HER2+ | None | None |
| 377640 | exon10:c.1454delinsTGTATT:p.Lys485fs | Frameshift | Rare/ Recurrent | 63 | HR+HER2- | None | None |
| 307956 | exon10:c.1508_1509delinsT:p.Lys503fs | Frameshift | Novel | 32 | HR+HER2- | None | Aunt and sister: BC*2; uncle: stomach cancer*1 |
| 49 5595 | exon10:c.1709_1712dup:p.Val572fs | Frameshift | Novel | 42 | HR+HER2- | None | Mother: OC |
| 551725 | exon10:c.1765_1769del:p.Lys589fs | Frameshift | Rare | 42 | HR+HER2- | None | Mother: BC |
| 404025 | exon10:c.1825C>T:p.Gln609Ter | Nonsense |  | 31 | HR+HER2- | None | Aunt: uterine cancer |
| 188091 | exon10:c.1901del:p. Ala634fs | Frameshift | Rare | 50 | HR+HER2- | None | None |
| 399334 | exon11:c.2806_2809del:p.Ala938fs | Frameshift |  | 29 | HR+HER2- | None | Mother: BC |
| 184833 | exon11:c.2806_2809del:p.Ala938fs | Frameshift |  | 26 | HR+HER2+ | OC | Sister: BC |
| 136117 | exon11:c.2808_2811del:p.Ala938Profs | Frameshift | Recurrent | 44 | HR+HER2- | None | Sister: BC; father: colon cancer |
| 434956 | exon11:c.2808_2811del:p.Ala938Profs | Frameshift | Recurrent | 43 | TNBC | None | None |
| 452220 | exon11:c.2808_2811del:p.Ala938Profs | Frameshift | Recurrent | 26 | HR+HER2- | None | None |
| 480107 | exon11:c.2870del:p.Asn957fs | Frameshift |  | 31 | HR+HER2+ | None | Grandfather: cholangiocarcinoma |
| 521674 | exon11:c.3109C>T:p.Gln1037Ter | Nonsense | Recurrent | 78 | HR+HER2+ | None | Daughter: BC |
| 447847 | exon11:c.3109C>T:p.Gln1037Ter | Nonsense | Recurrent | 50 | TNBC | None | Mother: BC; father: lung cancer |
| 334478 | exon11:c.3109C>T:p.Gln1037Ter | Nonsense | Recurrent | 36 | HR+HER2- | None | Aunt: BC*2 |
| 457461 | exon11:c.3109C>T:p.Gln1037Ter | Nonsense | Recurrent | 35 | HR+HER2- | None | Mother: OC |
| 331870 | exon11:c.3109C>T:p.Gln1037Ter | Nonsense | Recurrent | 32 | HR+HER2- | None | None |
| 331691 | exon11:c.3109C>T:p.Gln1037Ter | Nonsense | Recurrent | 30 | HR+HER2- | None | Grandmother, aunt, and sister: BC*7 |
| M2136413 | exon11:c.3195_3198del:p.Asn1066fs | Frameshift |  | 42 | HR+HER2- | None | None |
| 466778 | exon11:c.3195_3198del:p.Asn1066fs | Frameshift |  | 35 | HR+HER2- | None | None |
| 454257 | exon11:c.4149del:p.Leu1384fs | Frameshift | Novel | 41 | HR+HER2- | None | None |
| 446266 | exon11:c.4151del:p.Leu1384fs | Frameshift | Rare | 36 | HR+HER2- | None | Grandmother: stomach cancer |
| 468697 | exon11:c.4415_4418del:p.Lys1472fs | Frameshift |  | 36 | HR-HER2+ | None | None |
| 522449 | exon11:c.4459_4460insC:p.Lys1487fs | Frameshift | Novel | 53 | TNBC | None | None |
| 420389 | exon11:c.5164_5165del:p.Ser1722fs | Frameshift | Recurrent | 50 | HR+HER2- | None | None |
| 425622 | exon11:c.5164_5165del:p.Ser1722fs | Frameshift | Recurrent | 49 | HR+HER2- | None | None |
| 501773 | exon11:c.5164_5165del:p.Ser1722fs | Frameshift | Recurrent | 44 | HR+HER2- | None | Sister: BC*1 |
| 387900 | exon11:c.5237_5238insT:p.Asn1747Ter | Frameshift | Novel | 37 | HR+HER2- | None | Grandmother: BC*1 |
| 478289 | exon11:c.5242del:p.Ser1748fs | Frameshift | Rare | 41 | HR+HER2- | None | None |
| 449061 | exon11:c.5504_5505insA:p.Asn1835fs | Frameshift | Novel | 47 | HR+HER2- | None | None |
| 329213 | exon11:c.5645C>A:p.Ser1882Ter | Nonsense | Recurrent | 30 | HR+HER2- | None | None |
| 406753 | exon11:c.5645C>A:p.Ser1882Ter | Nonsense | Recurrent | 41 | HR+HER2- | None | None |
| M2873373 | exon11:c.5645C>A:p.Ser1882Ter | Nonsense | Recurrent | 40 | HR+HER2- | None | Mother: OC |
| 201640 | exon11:c.5717_5718del:p.Asn1906fs | Frameshift | Rare | 35 | HR+HER2- | None | None |
| 355249 | exon11:c.5718_5719del:p.Leu1908fs | Frameshift | Novel | 38 | HR+HER2+ | None | None |
| 109926 | exon11:c.5722_5723del:p.Leu1908fs | Frameshift | Recurrent | 53 | HR+HER2- | None | Mother and aunt: BC*2; sister: lung cancer*1 |
| 502449 | exon11:c.5722_5723del:p.Leu1908fs | Frameshift | Recurrent | 46 | HR+HER2- | None | Father: BC |
| 496666 | exon11:c.5722_5723del:p.Leu1908fs | Frameshift | Recurrent | 46 | HR+HER2- | None | None |
| 276198 | exon11:c.5722_5723del:p.Leu1908fs | Frameshift | Recurrent | 40 | HR+HER2- | None | Aunt: BC*1 and OC*1; sister: lung cancer*1 |
| 371448 | exon11:c.5771_5774del:p.lle1924fs | Frameshift |  | 52 | HR+HER2- | None | None |
| 524447 | exon11:c.5777_5784del:p.Ser1926fs | Frameshift | Novel | 35 | TNBC | None | None |
| 455526 | exon11:c.6405_6409del:p.Asn2135fs | Frameshift |  | 35 | HR+HER2+ | None | Sister: BC*1 |
| 475034 | exon11:c.6405_6409del:p.Asn2135fs | Frameshift |  | 31 | HR+HER2- | None | Aunt: BC*1 |
| 350124 | exon11:c.6415G>T:p.Glu2139Ter | Nonsense | Rare | 34 | HR+HER2+ | None | None |
| 221960 | exon11:c.6446_6447insTA:p.Lys2150fs | Frameshift | Novel | 45 | HR+HER2- | None | None |
| M2371800 | exon11:c.6448_6449insTA:p.Lys2150fs | Frameshift | Novel | 47 | HR+HER2- | None | None |
| 419313 | exon11:c.6466_6469del:p.Ser2156fs | Frameshift |  | 40 | TNBC | None | Sister: BC*1; brother: stomach cancer |
| 492796 | exon11:c.6591_6592del:p.Glu2198fs | Frameshift |  | 53 | TNBC | None | None |
| M1676061 | exon11:c.6699_6702dup:p.Met2235fs | Frameshift | Rare | 49 | HR+HER2- | None | None |
| 353289 | exon11:c.6699_6702dup:p.Met2235fs | Frameshift | Rare | 44 | HR+HER2- | None | Mother: BC |
| 219011 | exon14:c.7133C>G:p.Ser2378Ter | Nonsense |  | 49 | HR+HER2- | None | None |
| M2669512 | exon14:c.7392dup:p.Ala2465fs | Frameshift | Novel | 49 | HR+HER2- | None | Mother: BC |
| 419237 | exon17:c.7816dup:p.Asp2606fs | Frameshift | Rare | 29 | HR+HER2- | None | None |
| 490658 | exon17:c.7878G>A:p.Trp2626Ter | Nonsense |  | 55 | TNBC | None | None |
| 260574 | exon17:c.7878G>A:p.Trp2626Ter | Nonsense |  | 51 | HR+HER2- | PaC | Father: lung cancer; brother: colon cancer*1 |
| 275327 | exon18:c.8009C>T:p.Ser2670Leu | Missense |  | 40 | HR+HER2- | None | Aunt: BC*1 |
| 454154 | exon18:c.8009C>T:p.Ser2670Leu | Missense |  | 32 | HR+HER2- | None | Brother: colon cancer*1 |
| 448856 | exon18:c.8245C>T:p.Gln2749Ter | Nonsense | Rare | 53 | HR+HER2- | None | Mother: BC |
| 499983 | exon22_24:c.(8754+1_8755-1)_  (9256+1_9257-1) del | Deletion |  | 27 | TNBC | None | Father: PrC |
| 474451 | exon22_24 del | Deletion |  | 47 | HR+HER2- | None | None |
| 544064 | exon22:c.8818_8824del:p.Lys2940fs | Frameshift | Rare | 31 | HR+HER2- | None | Grandmother: BC*1 |
| 520887 | exon22:c.8890dup:p.Arg2964fs | Frameshift |  | 33 | HR+HER2- | None | None |
| 287362 | exon22:c.8951C>G:p.Ser2984Ter | Nonsense |  | 70 | TNBC | None | Aunt: BC*1 |
| 485290 | exon22:c.8954-2A>G | Splice site | Novel | 54 | HR+HER2+ | None | None |
| 461398 | exon22:c.8954-2A>G | Splice site | Novel | 41 | HR+HER2- | None | None |
| 481776 | exon23:c.8961_8964del:p.Ser2988fs | Frameshift |  | 44 | TNBC | None | None |
| 206728 | exon23:c.8961_8964del:p.Ser2988fs | Frameshift |  | 40 | HR+HER2+ | None | None |
| 395749 | exon23: c.9089_9090insA:p.Thr3033fs | Frameshift | Rare | 39 | HR+HER2- | None | Sister: BC*1 |
| 440826 | exon23: c.9089_9090insA:p.Thr3033fs | Frameshift | Rare | 30 | HR+HER2+ | None | None |
| 527131 | exon23:c.9097dup:p.Thr3033fs | Frameshift |  | 42 | HR+HER2- | None | Mother: BC |
| 374811 | exon23:c.9097dup:p.Thr3033fs | Frameshift |  | 29 | HR+HER2- | None | None |
| 437765 | exon24:c.9253_9254insA:p.Thr3085fs | Frameshift | Novel | 33 | HR+HER2- | None | None |
| 394995 | exon24:c.9253_9254insA:p.Thr3085fs | Frameshift | Novel | 29 | TNBC | None | Mother and aunt: BC*2 |
| M2796807 | exon25:c.9401del:p.Gly3134fs | Frameshift |  | 33 | HR+HER2- | None | None |
| 290864 | exon25:c.9401del:p.Gly3134fs | Frameshift |  | 30 | HR+HER2- | None | None |
| 219587 | exon25:c.9407_9411del:p.Thr3137fs | Frameshift | Novel | 50 | HR+HER2- | OC | None |
| 402397 | /; / | / |  | 26 | HR+HER2- | None | None |

**#*BRCA2* (NM_000059.3). Abbreviations: BC, breast cancer; OC, ovarian cancer; PaC, pancreatic cancer; PrC, prostate cancer.**

**Supplementary table 5. Distribution of non-*BRCA* P/LPVs in 40 patients.**

| **Patient ID** | **Nucleotide and amino acid change** | **Variant type** | **Variant status** | **Diagnosis age (years)** | **Molecular subgroup** | **Secondary cancer** | **Family history of cancer** |
| --- | --- | --- | --- | --- | --- | --- | --- |
| 514939 | ATM:exon32:c.4852C>T:p.Arg1618Ter | Nonsense |  | 36 | HR+HER2- | None | None |
| 394699 | ATM:exon48:c.6975+2T>C | Splice site |  | 55 | TNBC | Colon cancer | None |
| 538004 | ATM:exon58:c.8494C>T:p.Arg2832Cys | Missense |  | 34 | HR+HER2- | None | None |
| 446397 | BARD1:exon1:c.69_70delinsTCCGGG  AACGAGCCTCGTTCCGCGT:p.Ala25fs | Frameshift |  | 34 | HR+HER2+ | None | None |
| 448248 | BARD1:exon5:c.1350dup:p.Gly451fs | Frameshift | Novel | 39 | HR+HER2- | None | None |
| 542184 | BARD1:exon11:c.2020G>T:p.Gly674Ter | Nonsense |  | 56 | HR+HER2- | None | None |
| 545851 | BRIP1:exon10:c.1343G>A:p.Trp448Ter | Nonsense |  | 32 | HR+HER2- | None | None |
| 451897 | CDH1:exon12:c.1828C>T:p.Gln610Ter | Nonsense | Novel | 40 | HR+HER2- | None | Grandmother, father, and aunt: stomach cancer*3 |
| 530728 | CHEK2:exon3:c.417C>A:p.Tyr139Ter | Nonsense |  | 38 | HR+HER2- | Lung cancer | Grandfather: lung cancer*1r |
| 473336 | CHEK2:exon11:c.1116dup:p.Lys373fs | Frameshift |  | 32 | HR+HER2- | None | None |
| 439000 | CHEK2:exon11:c.1186del:p.Val397fs | Frameshift | Novel | 37 | HR+HER2- | None | None |
| 212287 | MRE11A:exon10:c.1090C>T:p.Arg364Ter | Nonsense |  | 43 | HR+HER2- | Gallbladder cancer | Father: liver cancer |
| 489149 | MSH6:exon4:c.2230dup:p.Glu744fs | Nonsense |  | 31 | TNBC | None | None |
| 534810 | MUTYH:exon3:c.312C>A:p.Tyr104Ter | Nonsense | Novel | 34 | HR+HER2+ | Thyroid cancer | None |
| 518950 | PALB2:exon4:c.535C>T:p.Gln179Ter | Nonsense | Rare | 53 | HR-HER2+ | None | Sister: BC*1 |
| 445028 | PALB2:exon4:c.595_596insA:p.Leu199fs | Frameshift | Rare | 31 | HR+HER2- | None | Father: PrC |
| 502454 | PALB2:exon4:c.1068del:p.Lys356fs | Frameshift | Novel | 68 | HR+HER2- | None | None |
| 371221 | PALB2:exon4:c.1240C>T:p.Arg414Ter | Nonsense |  | 56 | HR+HER2- | None | None |
| M2300039 | PALB2:exon4:c.1592del:p.Leu531fs | Frameshift |  | 43 | HR+HER2- | None | Uncle: lung cancer*1 |
| 449118 | PALB2:exon4:c.1675_1676delinsTG:  p.Gln559Ter | Missense |  | 35 | HR+HER2- | None | None |
| 513051 | PALB2:exon5:c.2257C>T:p.Arg753Ter | Nonsense |  | 48 | TNBC | None | None |
| 495370 | PALB2:exon8:c.2760dup:p.Gln921fs | Frameshift | Rare | 35 | HR+HER2- | Thyroid cancer | None |
| 385953 | PALB2:exon9:c.2968G>T:p.Glu990Ter | Nonsense |  | 64 | HR+HER2- | None | Sister: BC*1 |
| 287884 | PALB2:exon9:c.2996+1G>T | Splice site |  | 37 | HR+HER2- | None | Mother: BC |
| 471578 | PALB2:exon12:c.3256C>T:p.Arg1086Ter | Nonsense |  | 42 | HR+HER2+ | None | None |
| 389148 | PTEN:exon1:c.70G>T:p.Asp24Tyr | Missense |  | 41 | TNBC | None | None |
| 220446 | PTEN:exon5:c.277C>T:p.His93Tyr | Missense |  | 35 | HR+HER2- | None | None |
| 488060 | PTEN:exon6:c.500del:p.Thr167fs | Frameshift | Novel | 43 | HR+HER2- | None | None |
| M2879142 | RAD50:exon19:c.2983_2986del:p.Glu995fs | Frameshift |  | 33 | TNBC | None | None |
| 516387 | RAD51C:exon2:c.394dup:p.Thr132fs | Frameshift |  | 53 | TNBC | None | Mother: OC |
| 465240 | RAD51C:exon6:c.905-2A>C | Splice site |  | 55 | HR-HER2+ | None | None |
| 481231 | TP53:exon3:c.96+1G>A | Splice site |  | 27 | HR+HER2+ | None | Mother: BC, uncle: colon*1 cancer |
| 473044 | TP53:exon4:c.118del:p.Met40fs | Frameshift | Novel | 32 | HR+HER2+ | Stomach cancer | None |
| 408523 | TP53:exon4:c.301A>T:p.Lys101Ter | Nonsense | Novel | 24 | HR+HER2+ | None | None |
| 336957 | TP53:exon4:c.321del:p.Tyr107fs | Frameshift | Novel | 37 | HR-HER2+ | None | None |
| 314709 | TP53:exon5:c.473G>T:p.Arg158Leu | Missense |  | 41 | TNBC | None | None |
| 488959 | TP53:exon5:c.559+1G>A | Splice site |  | 33 | HR+HER2+ | None | None |
| 457077 | TP53:exon7:c.768_769del:p.Leu257fs | Frameshift | Rare | 36 | HR-HER2+ | None | None |
| 374064 | TP53:exon8:c.833C>T:p.Pro278Leu | Missense |  | 31 | HR-HER2+ | None | None |
| 481044 | TP53:exon8:c.916C>T:p.Arg306Ter | Nonsense |  | 39 | HR+HER2- | None | Father: liver cancer; aunt: BC; uncle: lung cancer |
| 396551 | TP53:exon9:c.994-1G>T | Splice site |  | 39 | HR+HER2- | None | None |

**Abbreviations: BC, breast cancer; OC, ovarian cancer; PaC, pancreatic cancer; PrC, prostate cancer.**

**Supplemental Table 6.** **List of variants of uncertain significance.**

| **Gene** | **Nucleotide and amino acid change** | **No. of Patient(s)** |
| --- | --- | --- |
| ***ATM* (N=44)** | exon1:c.-30-15G>A | 1 |
|  | exon2:c.72+4G>A | 1 |
|  | exon4:c.283C>A:p.Gln95Lys | 3 |
|  | exon7:c.745A>G:p.Ile249Val | 1 |
|  | exon8:c.1037T>A:p.Ile346Asn | 1 |
|  | exon10:c.1511A>G:p.Asn504Ser | 2 |
|  | exon14:c.2159G>A:p.Arg720His | 1 |
|  | exon15:c.2275A>G:p.Ser759Gly | 1 |
|  | exon16:c.2466+10C>G | 1 |
|  | exon17:c.2638+8C>T | 1 |
|  | exon18:c.2694A>C:p.Leu898Phe | 1 |
|  | exon18:c.2781G>T:p.Leu927Phe | 1 |
|  | exon19:c.2922-8T>C | 1 |
|  | exon23:c.3402+14A>C | 1 |
|  | exon25:c.3743A>G:p.Tyr1248Cys | 1 |
|  | exon26:c.3925G>A:p.Ala1309Thr | 2 |
|  | exon30:c.4611+11G>C | 1 |
|  | exon31:c.4618G>T:p.Asp1540Tyr | 1 |
|  | exon31:c.4667A>C:p.Tyr1556Ser | 1 |
|  | exon32:c.4909+6A>G | 1 |
|  | exon33:c.4916C>T:p.Pro1639Leu | 1 |
|  | exon36:c.5464G>A:p.Glu1822Lys | 1 |
|  | exon37:c.5628C>A:p.His1876Gln | 1 |
|  | exon38:c.5738T>G:p.Val1913Gly | 1 |
|  | exon39:c.5919-8A>G | 1 |
|  | exon40:c.5945A>G:p.Gln1982Arg | 1 |
|  | exon41:c.6058G>A:p.Gly2020Ser | 1 |
|  | exon42:c.6101G>A:p.Arg2034Gln | 2 |
|  | exon42:c.6173C>T:p.Ser2058Leu | 1 |
|  | exon42:c.6562C>A:p.Leu2188Ile | 1 |
|  | exon45:c.6497T>C:p.Val2166Ala | 1 |
|  | exon48:c.7064T>A:p.Val2355Asp | 1 |
|  | exon52:c.7739G>A:p.Arg2580Lys | 1 |
|  | exon52:c.7740A>C:p.Arg2580Ser | 1 |
|  | exon54:c.7949A>G:p.Asp2650Gly | 1 |
|  | exon55:c.8071C>T:p.Arg2691Cys | 3 |
|  | exon56:c.8246A>T:p.Lys2749Ile | 1 |
| ***BRCA2* (N=42)** | exon4:c.323A>G:p.Asn108Ser | 1 |
|  | exon5:c.443G>A:p.Cys148Tyr | 1 |
|  | exon7:c.599C>T:p.Thr200Ile | 1 |
|  | exon10:c.943 T>A:p.Cys315Ser | 1 |
|  | exon10:c.1162G>T:p.Val388Leu | 1 |
|  | exon10:c.1058C>T:p.Ser353Leu | 1 |
|  | exon10:c.1211A>G:p.Asn404Ser | 1 |
|  | exon10:c.1416G>C:p.Gln472His | 1 |
|  | exon10:c.1600G>A:p.Glu534Lys | 1 |
|  | exon10:c.1732G>C:p.Gly578Arg | 2 |
|  | exon11:c.2122T>A:p.Ser708Thr | 1 |
|  | exon11:c.2405A>G:p.Asn802Ser | 1 |
|  | exon11:c.2920G>A:p.Asp974Asn | 1 |
|  | exon11:c.3197A>G:p.Asn1066Ser | 1 |
|  | exon11:c.3220A>T:p.Ser1074Cys | 1 |
|  | exon11:c.3256A>G:p.Ile1086Val | 2 |
|  | exon11:c.3938A>G:p.Tyr1313Cys | 2 |
|  | exon11:c.5218_5223del:p.Leu1740_Ser1741del | 1 |
|  | exon11:c.5467A>G:p.Lys1823Glu | 1 |
|  | exon11:c.5554G>A:p.Val1852Ile | 1 |
|  | exon11:c.6076A>T:p.Thr2026Ser | 1 |
|  | exon14:c.7088A>G:p.Tyr2363Cys | 1 |
|  | exon14:c.7324T>G:p.Ser2442Ala | 1 |
|  | exon14:c.7418G>A:p.Cys2473Tyr | 1 |
|  | exon14:c.7426_7427delinsCC:p.Glu2476Pro | 1 |
|  | exon15:c.7522G>A:p.Gly2508Ser | 4 |
|  | exon16:c.7670C>T:p.Ala2557Val | 1 |
|  | exon19:c.8474C>A:p.Ala2825Glu | 1 |
|  | exon20:c.8633-16C>A | 1 |
|  | exon22:c.8798G>A:p.Arg2933Lys | 1 |
|  | exon23:c.9106C>G:p.Gln3036Glu | 2 |
|  | exon26:c.9538C>T:p.Leu3180Phe | 1 |
|  | exon27:c.9875C>T:p.Pro3292Leu | 1 |
|  | exon27:c.10024G>A:p.Glu3342Lys | 1 |
|  | exon27:c.10163C>A:p.Thr3388Lys | 1 |
| ***MUTYH* (N=34)** | exon2:c.53C>T:p.Pro18Leu | 10 |
|  | exon2:c.74G>A:p.Gly25Asp | 11 |
|  | exon3:c.179A>G:p.Glu60Gly | 1 |
|  | exon3:c.226G>A:p.Val76Ile | 1 |
|  | exon7:c.523G>T:p.Ala175Ser | 2 |
|  | exon8:c.551G>A:p.Arg184Gln | 1 |
|  | exon10:c.821G>A:p.Arg274Gln | 1 |
|  | exon10:c.934-2A>G | 5 |
|  | exon11:c.961G>A:p.Gly321Arg | 1 |
|  | exon11:c.998-9C>T | 1 |
| ***RAD50* (N=21)** | exon1:c.38G>A:p.Arg13Gln | 1 |
|  | exon1:c.88C>T:p.Pro30Ser | 1 |
|  | exon3:c.265G>A:p.Val89Ile | 2 |
|  | exon4:c.511G>T:p.Ala171Ser | 3 |
|  | exon7:c.1052-6dup | 1 |
|  | exon8:c.1094G>A:p.Arg365Gln | 1 |
|  | exon8:c.1211A>G:p.Gln404Arg | 1 |
|  | exon10:c.1635+6A>C | 1 |
|  | exon11:c.1688_1689delinsAG:p.Arg564Gln | 1 |
|  | exon13:c.2083C>G:p.Gln695Glu | 1 |
|  | exon15:c.2501A>G:p.Glu834Gly | 1 |
|  | exon16:c.2686A>G:p.Thr896Ala | 1 |
|  | exon16:c.2718+17A>G | 1 |
|  | exon17:c.2813A>G:p.Lys938Arg | 1 |
|  | exon23:c.3496C>T:p.Arg1166Trp | 1 |
|  | exon24:c.3620T>C:p.Val1207Ala | 1 |
|  | exon25:c.3836G>A:p.Arg1279His | 1 |
|  | exon25:c.3902A>G:p.Lys1301Arg | 1 |
| ***PMS1* (N=20)** | exon3:c.316-4C>T | 1 |
|  | exon3:c.265A>G:p.Thr89Ala | 1 |
|  | exon9:c.1162A>C:p.Ile388Leu | 1 |
|  | exon9:c.1492T>C:p.Trp498Arg | 1 |
|  | exon10:c.1981A>T:p.Asn661Tyr | 1 |
|  | exon10:c.1996C>G:p.His666Asp | 1 |
|  | exon11:c.2444C>T:p.Ala815Val | 1 |
|  | exon12:c.2635-10C>G | 1 |
|  | exon13:c.2755C>T:p.Arg919Cys | 10 |
|  | exon13:c.2780A>G:p.Tyr927Cys | 2 |
| ***CHEK2* (N=18)** | exon4:c.538C>T:p.Arg180Cys | 6 |
|  | exon4:c.542G>A:p.Arg181His | 2 |
|  | exon5:c.613A>T:p.Thr205Ser | 1 |
|  | exon6:c.755G>A:p.Ser252Asn | 2 |
|  | exon9:c.1008+3A>T | 1 |
|  | exon11:c.1111C>T:p.His371Tyr | 5 |
|  | exon12:c.1318A>G:p.Ile440Val | 1 |
| ***PALB2* (N=18)** | exon4:c.661G>A:p.Val221Ile | 1 |
|  | exon4:c.1213C>G:p.Pro405Ala | 4 |
|  | exon4:c.1327A>G:p.Lys443Glu | 1 |
|  | exon4:c.1660G>A:p.Glu554Lys | 1 |
|  | exon5:c.2129C>T:p.Thr710Met | 1 |
|  | exon5:c.2345C>A:p.Pro782Gln | 1 |
|  | exon5:c.2360C>T:p.Thr787Ile | 2 |
|  | exon5:c.2474G>C:p.Arg825Thr | 1 |
|  | exon5:c.2506G>C:p.Val836Leu | 1 |
|  | exon8:c.2815T>G:p.Leu939Val | 1 |
|  | exon10:c.3004G>A:p.Glu1002Lys | 1 |
|  | exon10:c.3035C>T:p.Thr1012Ile | 1 |
|  | exon10:c.3054G>C:p.Glu1018Asp | 1 |
|  | exon13:c.3351G>C:p.Arg1117Ser | 1 |
| ***BRIP1* (N=17)** | exon4:c.305A>G:p.Gln102Arg | 1 |
|  | exon6:c.569G>A:p.Gly190Glu | 1 |
|  | exon6:c.628-16C>A | 1 |
|  | exon7:c.656_658del:p.Cys219del | 1 |
|  | exon7:c.739T>C:p.Tyr247His | 1 |
|  | exon10:c.1442G>A:p.Gly481Asp | 3 |
|  | exon10:c.1474-16T>C | 2 |
|  | exon15:c.2103A>C:p.Leu701Phe | 1 |
|  | exon16:c.2324A>G:p.Asn775Ser | 2 |
|  | exon17:c.2440C>T:p.Arg814Cys | 1 |
|  | exon19:c.2830C>G:p.Gln944Glu | 2 |
|  | exon20:c.3533A>T:p.Glu1178Val | 1 |
| ***BARD1* (N=16)** | exon1:c.54C>G:p.Asn18Lys | 1 |
|  | exon1:c.127C>A:p.Arg43Ser | 1 |
|  | exon3:c.233G>A:p.Cys78Tyr | 1 |
|  | exon3:c.236T>C:p.Ile79Thr | 1 |
|  | exon4:c.863C>G:p.Ser288Cys | 1 |
|  | exon4:c.977A>G:p.Asn326Ser | 1 |
|  | exon4:c.1217G>A:p.Arg406Gln | 1 |
|  | exon6:c.1412A>T:p.His471Leu | 1 |
|  | exon6:c.1429G>A:p.Val477Met | 1 |
|  | exon6:c.1468A>G:p.Thr490Ala | 1 |
|  | exon6:c.1475A>T:p.Tyr492Phe | 1 |
|  | exon6:c.1479A>C:p.Gln493His | 1 |
|  | exon7:c.1586G>A:p.Arg529Gln | 2 |
|  | exon8:c.1694G>A:p.Arg565His | 1 |
|  | exon11:c.2180A>G:p.Asp727Gly | 1 |
| ***BRCA1* (N=16)** | exon5:c.302-6T>G | 1 |
|  | exon7:c.548-5T>C | 1 |
|  | exon7:c.548-9del | 1 |
|  | exon10:c.824G>A:p.Gly275Asp | 1 |
|  | exon10:c.2005A>C:p.Met669Leu | 1 |
|  | exon10:c.2387C>T:p.Thr796Ile | 1 |
|  | exon10:c.2833A>G:p.Ser945Gly | 1 |
|  | exon10:c.3257T>A:p.Leu1086* | 1 |
|  | exon10:c.3287A>G:p.Gln1096Arg | 1 |
|  | exon10:c.3596C>T:p.Ala1199Val | 3 |
|  | exon13:c.4387T>C:p.Tyr1463His | 1 |
|  | exon16:c.5068A>C:p.Lys1690Gln | 2 |
|  | exon19:c.5216A>C:p.Asp1739Ala | 1 |
| ***MSH6* (N=16)** | exon1:c.234A>C:p.Arg78Ser | 1 |
|  | exon2:c.263G>A:p.Cys88Tyr | 3 |
|  | exon4:c.1037C>G:p.Ser346Cys | 1 |
|  | exon4:c.1063G>A:p.Gly355Ser | 1 |
|  | exon4:c.1085C>G:p.Pro362Arg | 1 |
|  | exon4:c.2300C>G:p.Thr767Ser | 1 |
|  | exon4:c.2527A>G:p.Ile843Val | 1 |
|  | exon4:c.2540A>G:p.Glu847Gly | 1 |
|  | exon4:c.2615T>C:p.Ile872Thr | 1 |
|  | exon4:c.3101G>A:p.Arg1034Gln | 1 |
|  | exon8:c.3689C>G:p.Ala1230Gly | 2 |
|  | exon8:c.3762A>T:p.Glu1254Asp | 1 |
|  | exon10:c.4045G>A:p.Ala1349Thr | 1 |
| ***MSH2* (N=15)** | exon2:c.232G>A:p.Val78Ile | 2 |
|  | exon3:c.427G>C:p.Ala143Pro | 1 |
|  | exon6:c.1076+16A>C | 1 |
|  | exon7:c.1096T>C:p.Phe366Leu | 1 |
|  | exon7:c.1121A>G:p.Gln374Arg | 1 |
|  | exon9:c.1435A>C:p.Ser479Arg | 1 |
|  | exon9:c.1480T>C:p.Ser494Pro | 1 |
|  | exon9:c.1510+11G>C | 1 |
|  | exon14:c.2447A>G:p.Gln816Arg | 1 |
|  | exon14:c.2458+8C>G | 1 |
|  | exon15:c.2516A>G:p.His839Arg | 1 |
|  | exon16:c.2785C>T:p.Arg929Ter | 3 |
| ***PMS2* (N=15)** | exon2:c.71A>G:p.His24Arg | 1 |
|  | exon4:c.327A>C:p.Glu109Asp | 1 |
|  | exon5:c.452G>A:p.Arg151His | 1 |
|  | exon5:c.538-16T>G | 1 |
|  | exon6:c.598G>A:p.Val20lle | 2 |
|  | exon9:c.904G>T:p.Val302Phe | 1 |
|  | exon9:c.962T>C:p.Val321Ala | 1 |
|  | exon11:c.1582G>A:p.Gly528Ser | 1 |
|  | exon11:c.1642G>C:p.Asp548His | 1 |
|  | exon11:c.1688_1689delinsAG:p.Arg563Gln | 1 |
|  | exon11:c.1843G>A:p.Val615Ile | 1 |
|  | exon11:c.1928A>G:p.Gln643Arg | 1 |
|  | exon12:c.2081A>T:p.Asp694Val | 1 |
|  | exon14:c.2405G>A:p.Arg802Gln | 1 |
| ***MRE11A* (N=13)** | exon3:c.37T>C:p.Phe13Leu | 1 |
|  | exon4:c.260G>A:p.Arg87Gln | 1 |
|  | exon4:c.310A>T:p.Ser104Cys | 1 |
|  | exon6:c.469A>G:p.Met157Val | 3 |
|  | exon8:c.681C>G:p.Asn227Lys | 2 |
|  | exon11:c.1219A>C:p.Lys407Gln | 1 |
|  | exon13:c.1362G>A:p.Met454Ile | 1 |
|  | exon13:c.1480G>A:p.Glu494Lys | 1 |
|  | exon15:c.1724G>A:p.Gly575Asp | 1 |
|  | exon16:c.1796C>T:p.Thr599Ile | 1 |
| ***CDH1* (N=12)** | exon1:c.4G>C:p.Gly2Arg | 1 |
|  | exon3:c.244G>A:p.Val82Met | 1 |
|  | exon5:c.688-14C>T | 1 |
|  | exon6:c.823G>T:p.Ala275Ser | 1 |
|  | exon7:c.892G>A:p.Ala298Thr | 1 |
|  | exon8:c.1018A>G:p.Thr340Ala | 4 |
|  | exon10:c.1565C>T:p.Thr522Ile | 1 |
|  | exon12:c.1844T>C:p.Ile615Thr | 1 |
|  | exon15:c.2439+14G>A | 1 |
| ***NBN* (N=9)** | exon1:c.37+6G>A | 1 |
|  | exon2:c.105T>G:p.Ile35Met | 1 |
|  | exon5:c.505C>T:p.Arg169Cys | 1 |
|  | exon7:c.745G>C:p.Ala249Pro | 1 |
|  | exon9:c.1023C>G:p.Ser341Arg | 2 |
|  | exon9:c.1036G>A:p.Val346Met | 1 |
|  | exon10:c.1398-10T>A | 1 |
|  | exon14:c.2185-17T>C | 1 |
| ***TP53* (N=7)** | exon3:c.91G>A:p.Val31Ile | 1 |
|  | exon4:c.265C>A:p.Pro89Thr | 1 |
|  | exon4:c.375+16G>A | 2 |
|  | exon7:c.743G>A:p.Arg248Gln | 1 |
|  | exon10:c.1073A>T:p.Glu358Val | 1 |
|  | exon11:c.1168C>T:p.Pro390Thr | 1 |
| ***STK11* (N=7)** | exon1:c.265C>T:p.Pro89Ser | 1 |
|  | exon3:c.439C>T:p.Arg147Cys | 1 |
|  | exon3:c.464+11G>A | 1 |
|  | exon4:c.597+21dup | 1 |
|  | exon5:c.735-6_735-2del | 1 |
|  | exon8:c.1088C>T:p.Thr363Ile | 1 |
|  | exon9:c.1217C>A:p.Ala406Glu | 1 |
| ***MLH1* (N=7)** | exon9:c.776T>C:p.Leu259Ser | 1 |
|  | exon12:c.1136A>C:p.Tyr379Ser | 1 |
|  | exon12:c.1163C>G:p.Ser388Cys | 1 |
|  | exon13:c.1441A>G:p.Met481Val | 1 |
|  | exon15:c.1730C>T:p.Ser577Leu | 1 |
|  | exon16:c.1742C>T:p.Pro581Leu | 1 |
|  | exon17:c.1990-9T>C | 1 |
| ***PTEN* (N=3)** | exon1:c.80-11A>G | 1 |
|  | exon2:c.165-8_165-4del | 1 |
|  | exon5:c.493-14T>C | 1 |
| ***RAD51C* (N=2)** | exon2:c.233C>T: p.Thr78lle | 1 |
|  | exon6:c.905-18_905-16del | 1 |
